# First principles study on structural, electronic and optical properties of HfS_2(1−*x*)_Se_2*x*_ and ZrS_2(1−*x*)_Se_2*x*_ ternary alloys†

**DOI:** 10.1039/d2ra01905a

**Published:** 2022-05-11

**Authors:** Mohammadreza Razeghizadeh, Mahdi Pourfath

**Affiliations:** School of Electrical and Computer Engineering, College of Engineering, University of Tehran Tehran 14395-515 Iran pourfath@ut.ac.ir

## Abstract

Alloying 2D transition metal dichalcogenides (TMDs) with dopants to achieve ternary alloys is as an efficient and scalable solution for tuning the electronic and optical properties of two-dimensional materials. This study provides a comprehensive study on the electronic and optical properties of ternary HfS_2(1−*x*)_Se_2(*x*)_ and ZrS_2(1−*x*)_Se_2(*x*)_ [0 ≤ *x* ≤ 1] alloys, by employing density functional theory calculations along with random phase approximation. Phonon dispersions were also obtained by using density functional perturbation theory. The results indicate that both of the studied ternary families are stable and the increase of Selenium concentration in HfS_2(1−*x*)_Se_2(*x*)_ and ZrS_2(1−*x*)_Se_2(*x*)_ alloys results in a linear decrease of the electronic bandgap from 2.15 (ev) to 1.40 (ev) for HfS_2(1−*x*)_Se_2(*x*)_ and 1.94 (ev) to 1.23 (ev) for ZrS_2(1−*x*)_Se_2(*x*)_ based on the HSE06 functional. Increasing the Se concentration in the ternary alloys results in a red shift of the optical absorption spectra such that the main absorption peaks of HfS_2(1−*x*)_Se_2(*x*)_ and ZrS_2(1−*x*)_Se_2(*x*)_ cover a broad visible range from 3.153 to 2.607 eV and 2.405 to 1.908 eV, respectively. The studied materials appear to be excellent base materials for tunable electronic and optoelectronic devices in the visible range.

## Abstract 2

Adding Selenium to HfS_2_ and ZrS_2_ two-dimensional materials allows tuning the optical properties in a wide visible spectrum that can be used in various electronic and optical applications, including solar cells.

## Graphical abstract

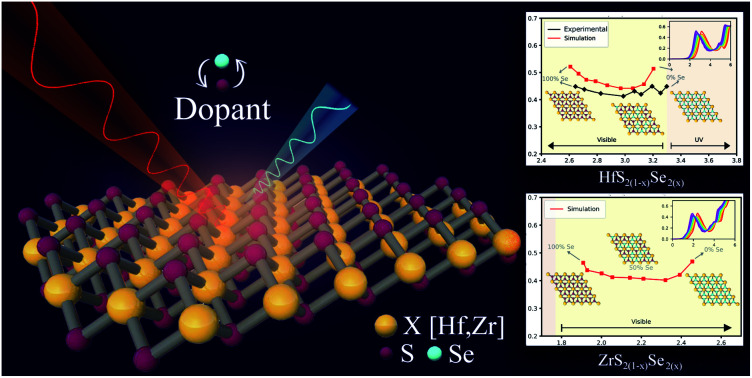

## 1 Introduction

The exploration of novel semiconductors with favorable electronic and optical properties^1,2^ is essential for the development of advanced optoelectronic devices, such as field effect transistors (FETs),^3^ photodetectors,^4^ phototransistors,^5^ solar cells,^6^ photovoltaics,^7^ memory devices,^8^ gas sensors,^9,10^ fiber lasers^11^ and photocatalysts.^7^ In recent years, two-dimensional (2D) materials have attracted the attention of many researchers, because of their rich physics and potential electronic and optoelectronic applications.^2,12^ Over the past decade, various 2D materials have been widely studied, including graphene,^13^ BN,^14^ transition metal dichalcogenides (TMDs or TMDCs)^15^ and phosphorene.^16^

Among 2D materials, TMDCs have been widely investigated^17^ due to the ease of fabrication,^18^ high carrier mobility^19^ and stability under environmental condition.^20^ The unit cell of a monolayer TMDC consists of one transition metal and two chalcogens.^15^ Each monolayer TMDC is made up of a set of three stacked layers: the lower chalcogen layer, the transition metal layer, and the upper chalcogen layer.^15^ 2D ternary TMDCs with the inclusion of a third element have shown tunable properties.^21–24^ Despite the availability of various approaches, such as mechanical strain, stacking in the form of heterostructure and applying electric field for energy bandgap engineering,^25,26^ alloying 2D TMDCs appear as an efficient and scalable solution.^27^ Ternary TMDCs, such as MoS_2(1−*x*)_Se_2*x*_,^28,29^ WS_2(1−*x*)_Se_2*x*_,^30^ ReS_2(1−*x*)_Se_2*x*_^31^ and MoSe_2*x*_Te_2(1−*x*)_,^32^ have shown distinct optical and electrical characteristics.

Hafnium dichalcogenides (HfX_2_, where X is a chalcogenide) are currently being studied by many groups, due to their interesting electronic and optical properties.^33–35^ Successful syntheses of HfX_2_ (X = S, Se) monolayers have been reported by using various techniques, including chemical vapor deposition,^33^ molecular beam epitaxy^36^ and ion beam-assisted process.^37^ Along with experimental studies, many theoretical investigations have been carried out to explore the properties of these materials. The electronic bandgaps of HfX_2_ (X = S, Se) in T-phase configuration are indirect^38^ with strong optical absorption peaks at 3.32 eV and 2.78 eV, respectively, for HfS_2_ and HfSe_2_.^33^ In contrast to other 2D materials, HfS_2_ has a much faster optical response.^39^ These excellent properties render HfS_2_ and HfSe_2_ monolayers as potential candidates for high performance electronic and optoelectronic applications such as photodetectors,^40^ phototransistors,^34^ solar cells,^41^ gas sensors^42^ and energy devices.^43^

Another focused material with fascinating properties in TMDC family is ZrX_2_ [X = S,Se].^44,45^ The electronic band structures of ZrX_2_ [X = S,Se] have been theoretically studied.^46,47^ On the experimental side, successful growth of ZrS_*x*_Se_2−*x*_ [0 ≤ *x* ≤ 2] has been reported.^48^ Zirconium dichalcogenides have been investigated for broadband visible light photodetection^49,50^ and solar-cell devices.^51^

By the use of alloying, the electronic^52,53^ and optical^21^ properties of HfX_2_ and ZrX_2_ [X = S,Se] can be engineered. The electronic and optical properties of ternary compounds can be tuned for new generation of photovoltaic devices. Recently, bandgaps and optical properties of HfS_2(1−*x*)_Se_2*x*_^33^ and ZrS_*x*_Se_2−*x*_^48^ were experimentally explored. In this work, a comprehensive theoretical study on the electronic, phononic, and optical properties of ternary HfS_2(1−*x*)_Se_2*x*_ and ZrS_2(1−*x*)_Se_2*x*_ [0 ≤ *x* ≤ 1] alloys is presented. The results are compared with available experimental data. Electronic bandstructure and bandgaps, dielectric functions, absorption coefficients, refractive indexes and optical conductivity of all ternary alloys are calculated, by using both PBE and HSE06 functionals. Presented results provide a deep insight into the alloying effects on electronic and optical properties of the studied materials pave the way for realizing tunable optoelectronic devices.

## 2 Theory and approach

The density functional theory (DFT) calculations as implemented in the Vienna *ab initio* simulation package (VASP)^54^ were employed in this study. The exchange-correlation energy was calculated based on generalized gradient approximation (GGA) as parametrized by Perdew–Burke–Ernzerhof (PBE).^55^ A plane-wave cutoff energy of 500 eV was selected. All the structures, including the atomic positions and cell parameters, were fully optimized with the conjugate gradient method until the total energy becomes smaller than 10^−5^ eV and the force on each atom becomes smaller than 0.01 eV Å^−1^. A 10 × 10 × 1 *k*-point mesh, generated by the Monkhorst–Pack scheme^56^ was used for the Brillouin-zone sampling in *k*-space for relaxing the structures. To isolate monolayers, a vacuum space of at least 17 Å was set along the out-of-plane direction to guarantee negligible interactions between adjacent layers. The phonon dispersion relation was analyzed to confirm the stability of the HfS_2_ and HfSe_2_ within a 4 × 4 × 1 supercell, by using the phonopy code^57^ interfaced with the density functional perturbation theory (DFPT)^58^ as implemented in VASP.

To obtain optical properties of ternary HfS_2(1−_*_x_*_)_Se_2_*_x_* and ZrS_2(1−_*_x_*_)_Se_2_*_x_*, the real and imaginary parts of the dielectric constants *ε*(*ω*) = *ε*_1_(*ω*) + *iε*_2_(*ω*) are evaluated. DFT calculations along with the random phase approximation (RPA)^59^ were used to evaluate the dielectric constants. A dense *k*-point mesh of 15 × 15 × 1 is used for the calculation of the imaginary part of frequency dependent dielectric function that is given by:^60^1
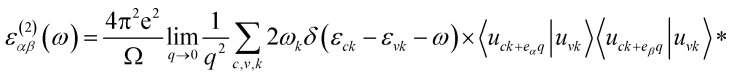
where the indices *α* and *β* are the Cartesian components, vectors *e*_*α*_ and *e*_*β*_ are the unit vectors along the respective component, *c* and *v* refer to conduction and valence band states, *ε*_*ck*_ and *ε*_*vk*_ are the band-edge energies of conduction and valence bands and *u*_*ck*_ is the cell periodic part of the orbitals at some wave-vector *k*. The real part of the dielectric function is obtained by the Kramers–Kronig transformation as:^60^2
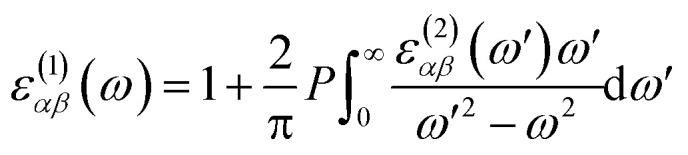


**Fig. 1:**
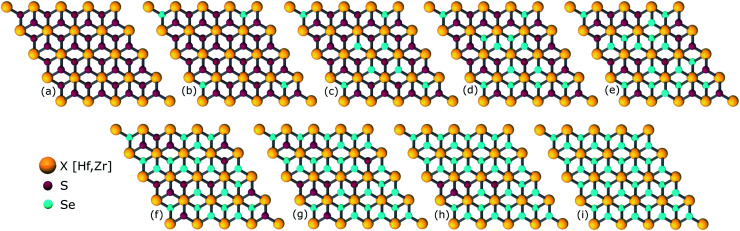
Atomic structures of ternary alloys (a) X_16_S_32_, (b) X_16_S_28_Se_4_, (c) X_16_S_24_Se_8_, (d) X_16_S_20_Se_12_, (e) X_16_S_16_Se_16_, (f) X_16_S_12_Se_20_, (g) X_16_S_8_Se_24_(h), X_16_S_4_Se_28_ and (i) X_16_Se_32_ [X = Hf,Zr].

**Fig. 2:**
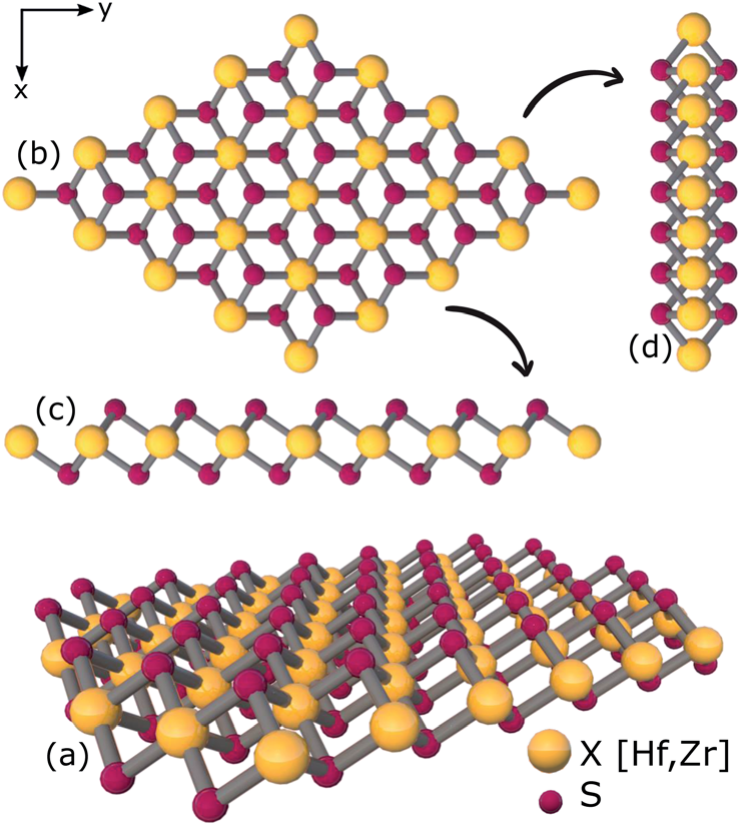
(a) 3D view (b) top view (c), (d) side views of relaxed structure of a 4 × 4 × 1 supercell of XS_2_ [X = Hf,Zr].

The study of ternary XS_2(1−*x*)_Se_2*x*_ [X = Hf,Zr] structures initiated by considering a 4 × 4 × 1 supercell of XS_2_ [X = Hf,Zr] monolayer that contains 16 X [X = Hf,Zr] and 32 S atoms (Fig. 2). Thereafter, 4, 8, 12, 16, 20, 24, 28, 32 S atoms were substituted with Se atoms as shown in Fig. 1. Various positions of Se atoms were carefully analyzed. The results indicate the position of the Se atoms does not affect the electronic and optical properties of the material. Therefore, S atoms were randomly substituted with Se atoms to obtain the desired ternary structure.

Due to the difference in the Zr–S and Zr–Se bond lengths in ZrS_2_ and ZrSe_2_ and Hf–S and Hf–Se bond lengths in HfS_2_ and HfSe_2_, ZrS_2(1−*x*)_Se_2*x*_ and HfS_2(1−*x*)_Se_2*x*_ alloys host an intrinsic lattice strain^61^ that yield a stronger modification of the electronic and optical properties.

## 3 Results and discussion

The impact of alloying XS_2_ [X = Hf,Zr] monolayer with Se atoms on the electronic and optical properties of ternary XS_2(1−*x*)_Se_2(*x*)_ [X = Hf,Zr] alloys are presented and discussed next.

### 3.1 Structural and phonon properties

**Fig. 3:**
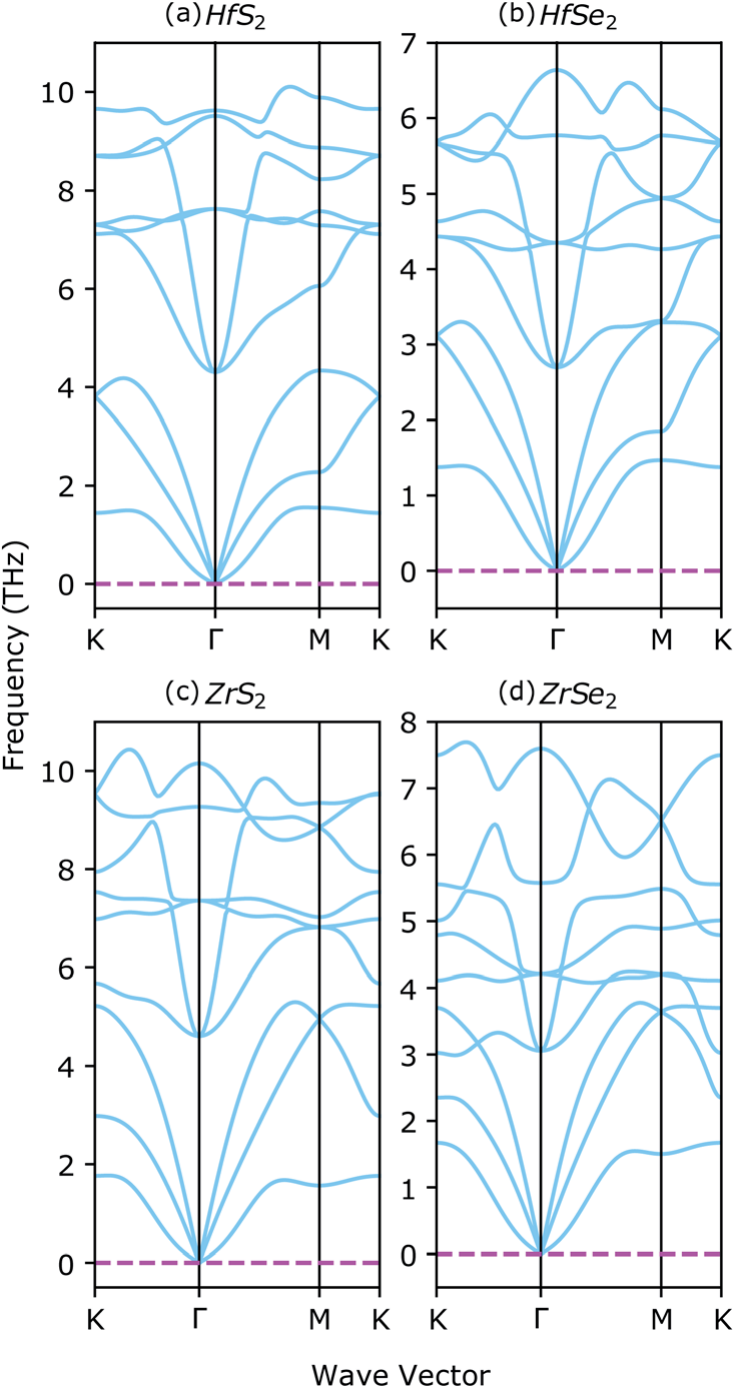
Phonon dispersion of (a) 1T-HfS_2_, (b) 1T-HfSe_2_, (c) 1T-ZrS_2_, and (d) 1T-ZrSe_2_ monolayers.

The stability of XS_2_, XSe_2_ and ternary XS_2(1−*x*)_Se_2(*x*)_ alloys [X = Hf,Zr] are proved in several theoretical and experimental studies.^33,36,37,62^ Here, vibrational properties of XS_2_ and XSe_2_ [X = Hf,Zr] are analyzed to confirm the structural stability. By using density functional perturbation theory (DFPT), the phonon dispersion around the high symmetry points of the BZ are calculated and illustrated in Fig. 3. The absence of negative frequencies in phonon dispersion is a strong indication of the stability of the studied materials. There are three acoustic and six optical branches in the four investigated compounds, because the unit cells include three atoms. By increasing the chalcogenide's atomic number from XS_2_ to XSe_2_ [X = Hf,Zr], the energies of optical branches are reduced. The acoustic branches around the high symmetry points of the BZ from the XS_2_ to XSe_2_ [X = Hf,Zr] are not affected, see Fig. 3(a)–(b) and Fig. 3(c)–(d).

### 3.2 Electronic properties

**Fig. 4:**
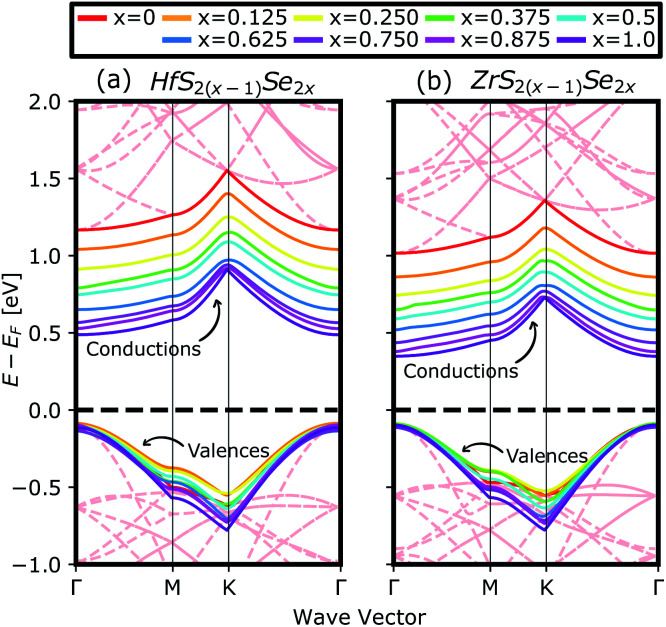
The calculated valence and conduction bands of (a) HfS_2(1−*x*)_Se_2(*x*)_ and (b) ZrS_2(1−*x*)_Se_2(*x*)_ ternary alloys, by using PBE functional. The red dashed lines in the background show the undoped band structures of 4 × 4 × 1 super cells of HfS_2_ and ZrS_2_. The zero-energy point was set at the Fermi-level.

**Fig. 5:**
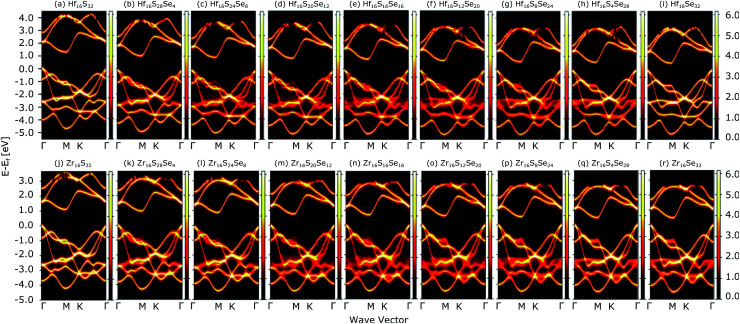
Unfolded electronic band-structures of HfS_2(1−*x*)_Se_2*x*_ ((a)–(i)) and ZrS_2(1−*x*)_Se_2*x*_ ((j)–(r)) ternary alloys. The zero energy point was set at the Fermi-level.

Fig. 4(a) compares the valence and conduction bands of HfS_2(1−*x*)_Se_2(*x*)_ and ZrS_2(1−*x*)_Se_2(*x*)_ with that of HfS_2_ and ZrS_2_ along the high symmetry paths of the BZ, calculated based on the PBE hybrid functional for *x* = 0, 0.125, 0.25, 0.375, 0.5, 0.625, 0.75, 0.875 and 1. The electronic bandgaps for both studied material families become smaller as the concentration of impurities increase in the HfS_2_ and ZrS_2_ mono-layers. Supercells are employed to study minor crystal structure modifications, such as the introduction of defects, distortions, and dopants.^63^ In such cases, it is helpful to determine how the original electronic structure is preserved.^63,64^ For this aim, unfolding the band structure of the supercell to the primitive cell is essential. The KPROJ^64^ package was used to unfold the band structure of the 4 × 4 × 1 supercells (Fig. 1). Fig. 5(a)–(i) and 5(j)–(r) show the unfolded electronic bandstructures of ternary HfS_2(1−*x*)_Se_2(*x*)_ and ZrS_2(1−*x*)_Se_2(*x*)_, respectively. Also, folded band structures of all studied ternary alloys are plotted in Fig. S1 and S2 of the ESI.†

**Fig. 6:**
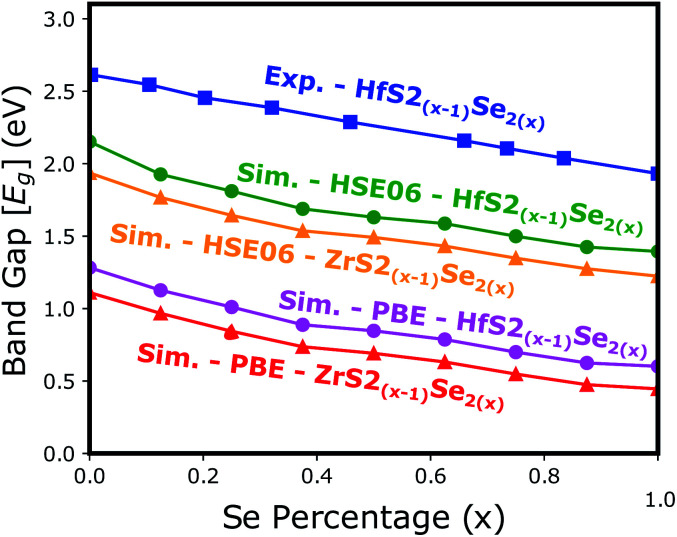
The electronic bandgap variations of HfS_2(1−*x*)_Se_2(*x*)_ and ZrS_2(1−*x*)_Se_2(*x*)_ as functions of Se atoms concentration.

Electronic band structure calculations based on HSE06 is more accurate than that by the PBE exchange-correlation energy,^28^ but HSE06 is computationally much more demanding. PBE approach underestimates the bandgap values, see Fig. 6, however, the trend of results is in good agreement with the experimental data.^33^ Furthermore, experimental and *ab initio* calculations show that the bandgap varies nearly linearly with the composition parameter *x* for both HfS_2(1−*x*)_Se_2(*x*)_ and ZrS_2(1−*x*)_Se_2(*x*)_ alloys.^33,48^ The calculated bandgaps, based on PBE and HSE06 functionals, along the lattice vectors of HfS_2(1−*x*)_Se_2(*x*)_ and ZrS_2(1−*x*)_Se_2(*x*)_ monolayers are reported in Table 1. Fig. 5 reveal that the valence band maximum (VBM) and the conduction band minimum (CBM) are located at the *Γ*- and *M*-points of the BZ for all HfS_2(1−*x*)_Se_2(*x*)_ and ZrS_2(1−*x*)_Se_2(*x*)_ alloys, respectively. Indicating that alloying HfS_2_ and ZrS_2_ with Se atoms does not change the nature of the bandgap and they remain indirect. With the increase of Se dopants both VBM and CBM move toward each other for both studied alloys, which results in the decrease of the electronic bandgap.

**Table 1:** Calculated relaxed lattice vectors (*a*,*b*) and electronic bandgap values for optimized HfS_2(1−*x*)_Se_2(*x*)_ and ZrS_2(1−*x*)_Se_2(*x*)_ monolayers

Material	Optimized lattice vectors (*a* = *b*) [Å]	Bandgap PBE [eV]	Bandgap HSE06 [eV]
HfS_2_	3.65	1.28	2.15
HfS_2(1−0.125)_Se_2(0.125)_	3.66	1.13	1.93
HfS_2(1−0.25)_Se_2(0.25)_	3.68	1.01	1.81
HfS_2(1−0.375)_Se_2(0.375)_	3.69	0.88	1.69
HfS_2(1−0.50)_Se_2(0.50)_	3.71	0.84	1.63
HfS_2(1−0.625)_Se_2(0.625)_	3.72	0.78	1.59
HfS_2(1−0.75)_Se_2(0.75)_	3.74	0.69	1.50
HfS_2(1−0.875)_Se_2(0.875)_	3.75	0.62	1.44
HfSe_2_	3.77	0.60	1.40
ZrS_2_	3.68	1.11	1.94
ZrS_2(1−0.125)_Se_2(0.125)_	3.70	0.97	1.77
ZrS_2(1−0.25)_Se_2(0.25)_	3.71	0.84	1.64
ZrS_2(1−0.375)_Se_2(0.375)_	3.72	0.74	1.54
ZrS_2(1−0.50)_Se_2(0.50)_	3.74	0.69	1.49
ZrS_2(1−0.625)_Se_2(0.625)_	3.75	0.63	1.43
ZrS_2(1−0.75)_Se_2(0.75)_	3.77	0.54	1.35
ZrS_2(1−0.875)_Se_2(0.875)_	3.78	0.47	1.28
ZrSe_2_	3.80	0.45	1.23

**Fig. 7:**
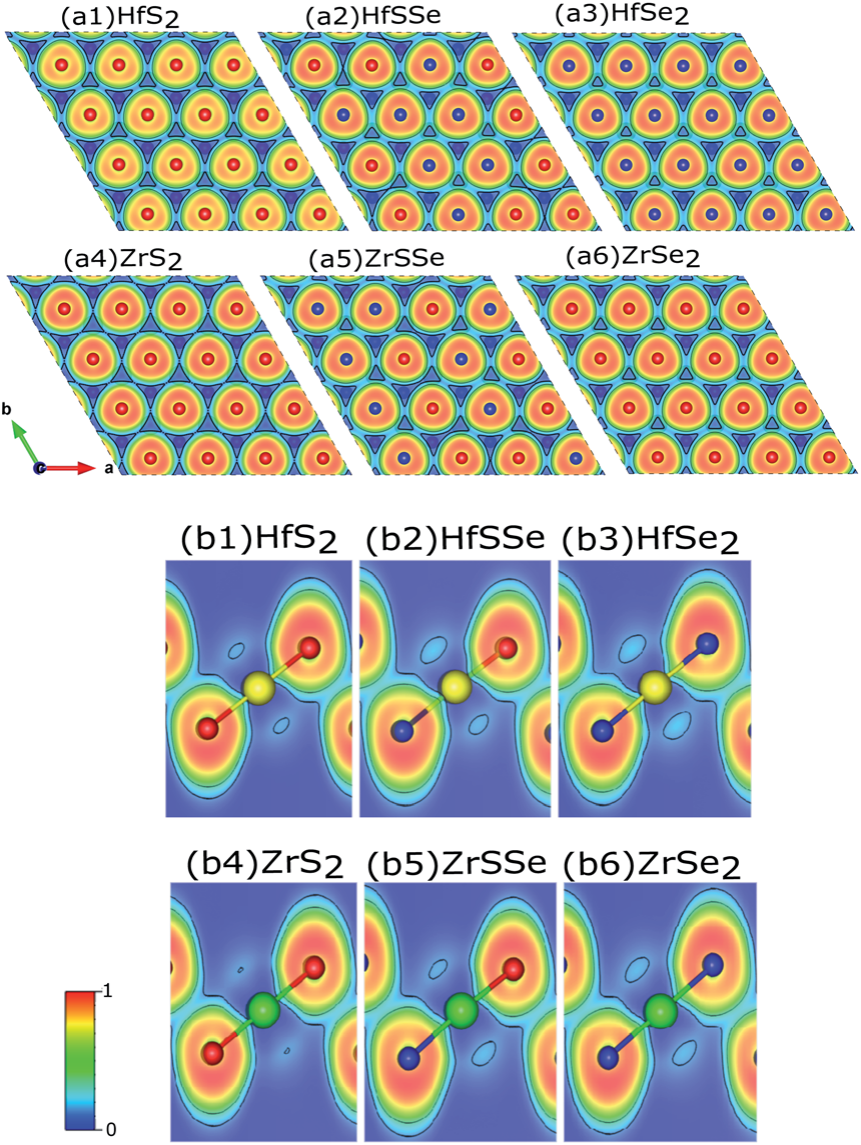
Cross sections of the electron localized function (ELF) of ternary mono-layers: (a1)–(a6) top views and (b1)–(b6) side views. Yellow, green, red and blue atoms represent Hf, Zr, S and Se respectively. Fig. (b1)–(b6) show that the Hf–S and Hf–Se bonds are covalent.

To discover the nature of the bandgap narrowing caused by the increase of selenium concentration, Bader charge, local electron function (ELF) and partial density of states (PDOS) analyses were performed. Selenium atomic radius is larger than that of sulfur and its electro-negativity is slightly smaller than that of sulfur that result in a larger bond length for Hf–Se bond. In the studied materials bond lengths for Hf–S and Hf–Se are 2.54 Å and 2.67 Å, respectively. A larger bond length along with a lower electro-negativity of selenium compared to sulfur, result in larger electron transfer between selenium and hafnium rather than sulfur. To ensure this, ELF of XS_2_, XSSe and XSe_2_ [X = Hf,Zr] are depicted in Fig. 7(a) and (b). As illustrated in Fig. 7(b1–b6) the X–S and X–Se [X = Hf,Zr] bonds are of ionic-covalent type, but the degree of covalent bonding is larger in X–Se [X = Hf,Zr] bond. As the electron attraction for Se atoms is smaller than that of S atoms, a larger electron cloud is observed around Se (Fig. 7(a1–a6)). Bader charge analysis of XS_2_, XSSe and XSe_2_ [X = Hf,Zr] (Table 2) indicate that charge losses of each X (Hf,Zr) atom and the gain of surrounding S and Se atoms for studied alloys. To relate the discussed charge transfer to the reduction of the bandgap, the contribution of orbitals to the bandstructure through PDOS is carefully investigated.

**Table 2:** Bader charge of the atomic sites in the XS2, XSSe and XSe2 [X = Hf,Zr] structures

Material	Hf	S	Se
HfS_2_	+30.114	−30.114	—
HfSSe	+29.38	−15.56	−13.82
HfSe_2_	+32.31	—	−32.31
ZrS_2_	+30.04	−30.04	—
ZrSSe	+28.91	−15.29	−13.62
ZrSe_2_	+32.04	—	−32.04

Both the total density of states (TDOS) and PDOS of studied alloys are shown in Fig. S3 and S4† of the supplementary, in which the significant contribution of the S, Se and Hf to the valence-band and conduction-band states of HfS_2(1−*x*)_Se_2(*x*)_ and contribution of the S, Se and Zr to the valence-band and conduction-band states of ZrS_2(1−*x*)_Se_2(*x*)_ can be observed. The PDOS of HfS_2(1−*x*)_Se_2(*x*)_ reveals that there is a strong hybridization between S(p), Se(p) and Hf(d) states. The top of the valence bands is mainly due to the contributions of S(p) while the bottom of the conduction band is mainly dominated by Hf(d) states. As selenium transfers more electrons from Se(p) to Hf(d) states than sulfur (Fig. 7(a)), the energy of Hf(d) states decreases with the increase of selenium concentration which in turn reduces the bandgap as illustrated in Fig. S3(a)–(i)† of the supplementary. Similar discussion holds for ZrS_2(1−*x*)_Se_2(*x*)_ alloys, see Fig. S4(a)–(i)† of the supplementary.

**Fig. 8:**
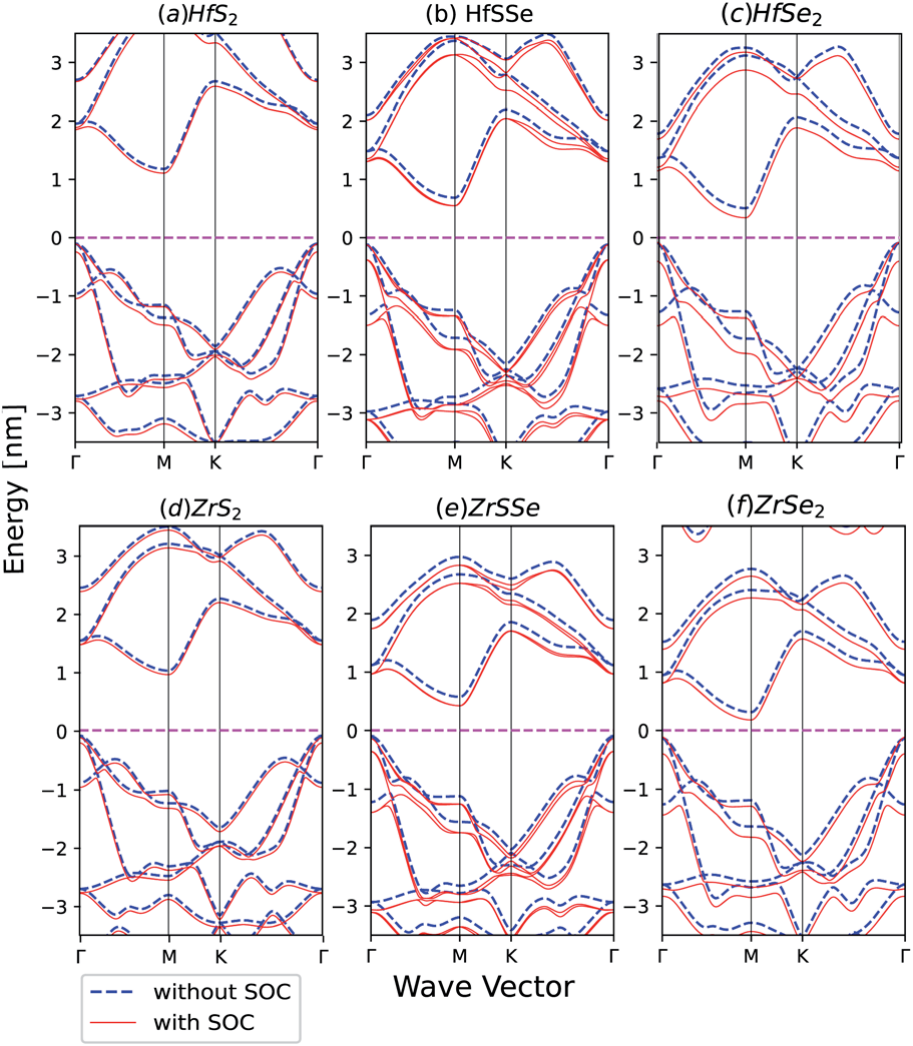
Band structures of (a) HfS_2_ (b) HfSSe (c) HfSe_2_ (d) ZrS_2_ (e) ZrSSe (f) ZrSe_2_ with and without spin–orbit coupling in the unit cell.

Spin–orbit coupling (SOC) effect on electronic band structures of HfS_2(1−*x*)_Se_2(*x*)_ and ZrS_2(1−*x*)_Se_2(*x*)_ monolayers is illustrated in Fig. 8(a)–(f). SOC has the least influence on HfS_2_ and ZrS_2_ electronic band-structures. As the selenium concentration increases, SOC lowers the bandgaps of HfS_2(1−*x*)_Se_2(*x*)_ and ZrS_2(1−*x*)_Se_2(*x*)_ ternary alloys. Furthermore, when the Se concentration increases, spin–orbit splitting in the highest valence band becomes larger in both studied ternary alloys. The bands in ZrX_2_ and HfX_2_ [X = S,Se] monolayers are degenerate due to inversion symmetry, whereas in XSSe [X = Hf,Zr] monolayers bands are no longer degenerate due to the lack of inversion symmetry.

### 3.3 Optical properties

**Fig. 9:**
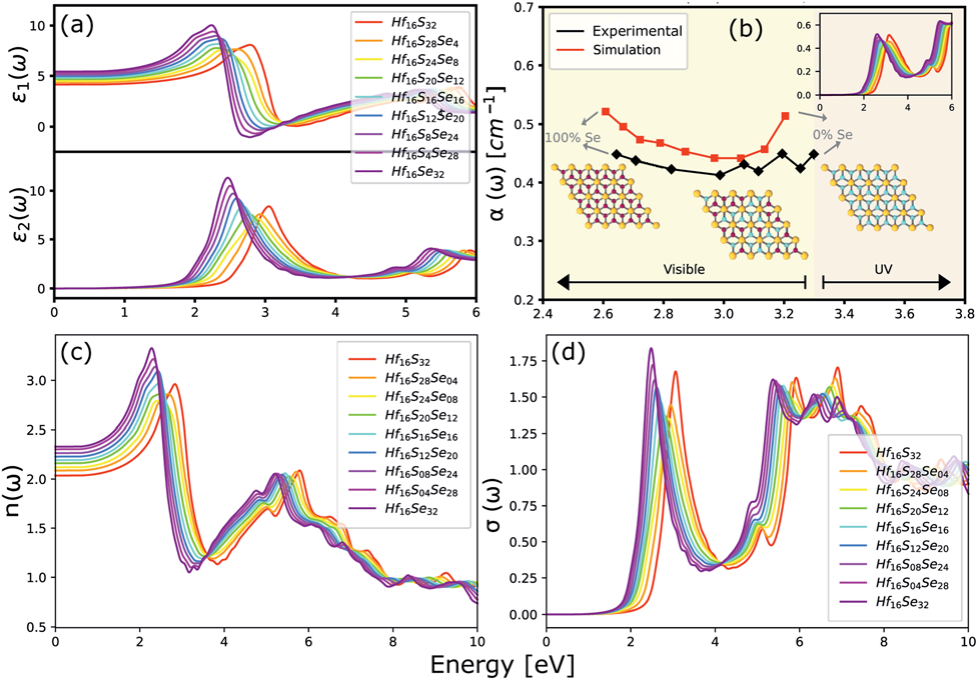
(a) The real and imaginary parts of the dielectric function. (b) The comparison between experimental (black line)^33^ and calculated absorption coefficients (red line). (c) The refractive index and (d) the optical conductivity for HfS_2(1−*x*)_Se_2(*x*)_ ternary alloys.

**Fig. 10:**
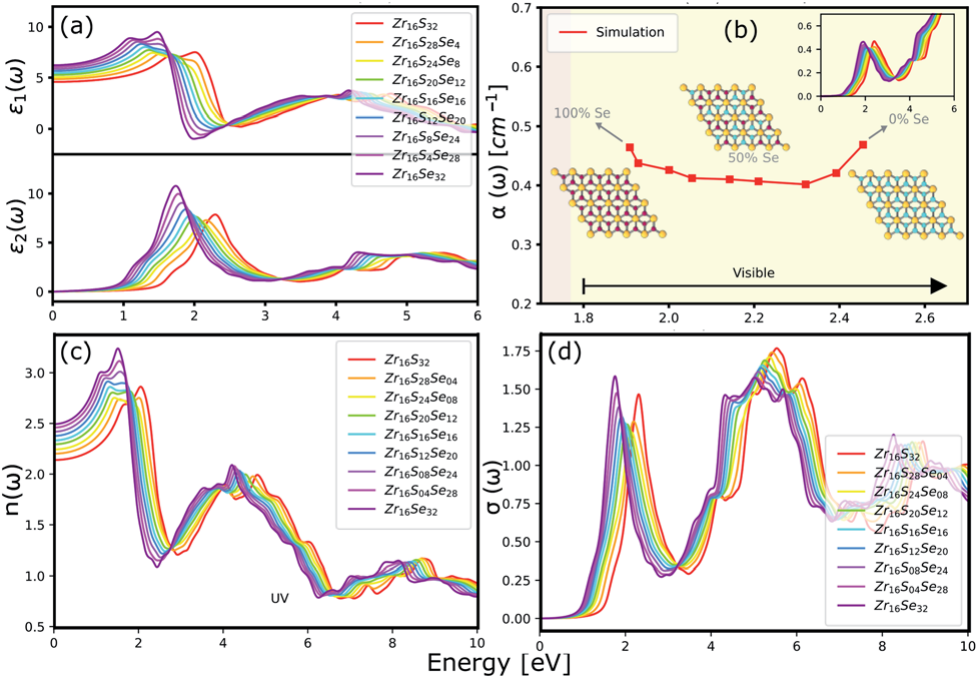
(a) The real and imaginary parts of the dielectric function. (b) The calculated absorption coefficients. (c) The refractive index and (d) the optical conductivity for ZrS_2(1−*x*)_Se_2(*x*)_ ternary alloys.

The optical properties of a material is accurately described by the complex dielectric function. Here, the imaginary part of dielectric function (*ε*_2_) is evaluated based on eqn (1), by using RPA in DFT calculations. Although RPA underestimates the screening in terms of dielectric function magnitude, this is often canceled by the PBE exchange-correlation underestimation of the bandgap.^65^ The real part (*ε*_1_) of the complex dielectric function is related to the imaginary part through the Kramers–Kronig relation (eqn (2)). Fig. 9(a) and 10(a) illustrate the real and imaginary parts of the complex dielectric functions for ternary HfS_2(1−*x*)_Se_2(*x*)_ and ZrS_2(1−*x*)_Se_2(*x*)_ alloys, respectively. The main peak of the imaginary part of HfS_2_ appears around 3.05 eV and is red-shifted by the substitutions of S atoms with Se dopants that can be explained by the reduction of the bandgap. Similarly, the peaks of ZrS_2(1−*x*)_Se_2(*x*)_ redshifts from 2.30 eV (*x* = 0) to 1.74 eV (*x* = 1).

The optical absorption and refractive index can be calculated from the complex dielectric function:^66,67^3
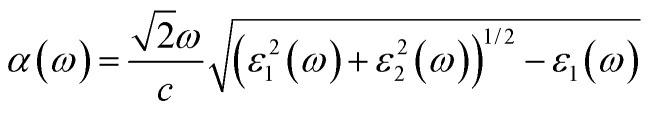
4
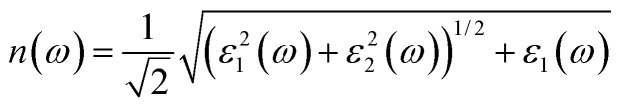


The calculated optical absorption coefficients and refractive indices of HfS_2(1−*x*)_Se_2(*x*)_ alloys are illustrated in Fig. 9(b) and (c), indicating that they cover a broad visible range from 3.153 to 2.607 eV. The calculated absorption coefficients are in good agreement with experimental results^33^ (Fig. 9(b)) that validates the accuracy of the utilized approach. The optical absorption coefficients as well as refractive indices of ZrS_2(1−*x*)_Se_2(*x*)_ are depicted in Fig. 10(a) and (b). The absorption peaks of ZrS_2(1−*x*)_Se_2(*x*)_ alloys are located in the visible range as well, but in contrast to HfS_2(1−*x*)_Se_2(*x*)_ alloys, they occur at lower energies: from 2.405 eV to 1.908 eV. Very good agreement of calculated absorption peak energies for studied alloys and available experimental data (Table 3) verifies the accuracy of the employed methodology.

**Table 3:** The comparison of the calculated and experimentally observed energies (in eV) of the optical absorption peaks of HfS_2(1−*x*)_Se_2(*x*)_ and ZrS_2(1−*x*)_Se_2(*x*)_ ternary alloys

Material	This study	Exp. in ref. 33,48
HfS_2_	3.153	3.323
HfS_2(1−0.125)_Se_2(0.125)_	3.135	3.279
HfS_2(1−0.25)_Se_2(0.25)_	3.056	3.218
HfS_2(1−0.375)_Se_2(0.375)_	2.968	3.089
HfS_2(1−0.50)_Se_2(0.50)_	2.872	3.092
HfS_2(1−0.625)_Se_2(0.625)_	2.788	3.012
HfS_2(1−0.75)_Se_2(0.75)_	2.722	2.851
HfS_2(1−0.875)_Se_2(0.875)_	2.666	2.719
HfSe_2_	2.607	2.782
ZrS_2_	2.405	2.410
ZrS_2(1−0.125)_Se_2(0.125)_	2.392	—
ZrS_2(1−0.25)_Se_2(0.25)_	2.320	—
ZrS_2(1−0.375)_Se_2(0.375)_	2.210	—
ZrS_2(1−0.50)_Se_2(0.50)_	2.142	2.150
ZrS_2(1−0.625)_Se_2(0.625)_	2.054	—
ZrS_2(1−0.75)_Se_2(0.75)_	2.000	1.970
ZrS_2(1−0.875)_Se_2(0.875)_	1.928	—
ZrSe_2_	1.908	1.900

The optical conductivity is another convenient parameter to study the optical response of materials. The optical conductivity (*σ*) is given by:^68^5
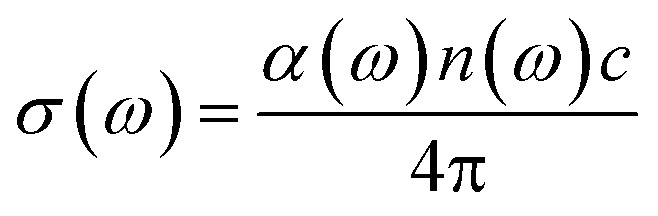


The optical conductivity directly depends on the absorption coefficient (*α*(*ω*)) and the refractive index (*n*(*ω*)) of the material, see Fig. 9(d) and 10(d). The first peaks of the optical conductivity appear at 2.45–3.10 eV (HfS_2(1−*x*)_Se_2(*x*)_) and 1.76–2.31 eV (ZrS_2(1−*x*)_Se_2(*x*)_). The optical absorption in a wide range of visible spectrum implies potential applications of HfS_2(1−*x*)_Se_2(*x*)_ and ZrS_2(1−*x*)_Se_2(*x*)_ layers for novel tunable electronic and optoelectronic devices. In another word, the ability to tune the bandgap and absorption spectrum with the Se concentration, render the studied materials as excellent candidates for third generation broadband photodetectors and solar cells.^22,69,70^

## 4 Conclusion

In summary, a comprehensive study on the structural, electronic and optical properties of ternary HfS_2(1−*x*)_Se_2(*x*)_ and ZrS_2(1−*x*)_Se_2(*x*)_ [0 ≤ *x* ≤ 1] alloys is presented, by employing first-principle calculations. It is shown that alloying is an efficient way for tuning electronic and optical properties of HfS_2_ and ZrS_2_ monolayers. In agreement with experimental studies, it is shown that both studied alloys are stable since no negative frequencies are observed in phonon dispersions. The HSE06 and PBE calculated electronic bandgaps for both of HfS_2(1−*x*)_Se_2(*x*)_ and ZrS_2(1−*x*)_Se_2(*x*)_ ternary alloys stay indirect while the bandgap linearly decrease as the Selenium concentration increases. Bader charge, ELF and DOS analyses indicate that the bandgap narrowing caused by increase of selenium in ternary alloys that originate from the difference in the electro-negativity and bond lengths of sulfur and selenium. As the selenium concentration increase, the conduction band decreases and the bandgap is reduced as well. Spin–orbit coupling has a negligible effect on XS_2_ [X = Hf,Zr], but as the selenium concentration increases in ternary alloys, SOC results in further bandgap reduction of HfS_2(1−*x*)_Se_2(*x*)_ and ZrS_2(1−*x*)_Se_2(*x*)_ ternary alloys. Optical properties of both studied ternary materials such as absorption, refractive index and conductivity red shift with the increase of selenium concentration that can be used as a tuning method. The absorption peaks of HfS_2(1−*x*)_Se_2(*x*)_ and ZrS_2(1−*x*)_Se_2(*x*)_ cover a broad visible range: from 3.153 to 2.607 eV and 2.405 to 1.908 eV, respectively. The studied alloys are excellent and potential candidates for novel tunable electronic and optoelectronic devices in various applications such as photodetectors, gas sensors and solar cells.

## Author contributions

Mahdi Pourfath contributed supervision and discussion.

## Conflicts of interest

None.

## Supplementary Material

RA-012-D2RA01905A-s001
