# Supplementary material for: First principles study on structural, electronic and optical properties of HfS2(1−x)Se2x and ZrS2(1−x)Se2x ternary alloys
Source: RSC Adv. 2022 May 11;12(22):14061–8. doi: 10.1039/d2ra01905a (PMC9092027; doi:10.1039/d2ra01905a)
Supplement: RA-012-D2RA01905A-s001 [file RA-012-D2RA01905A-s001.pdf]

Electronic Supplementary Material (ESI) for RSC Advances.
This journal is © The Royal Society of Chemistry 2022

Electronic Supplementary Material (ESI) for RSC Advances

First Principles Study on Structural, Electronic and Optical Properties of $HfS_{2(1-x)}Se_{2x}$ and $ZrS_{2(1-x)}Se_{2x}$ Ternary Alloys

Mohammadreza Razeghizadeh [a], Mahdi Pourfath [a,b,c,*]

[a] School of Electrical and Computer Engineering, University of Tehran, Tehran 14395-515, Iran.
[b] Super Computing Institute, University of Tehran, Tehran 14398-17435, Iran.
[c] Institute for Microelectronics, Technische Universität Wien, Vienna A-1040, Austria.
[*] Corresponding author. E-mail: pourfath@ut.ac.ir , pourfath@iue.tuwien.ac.at

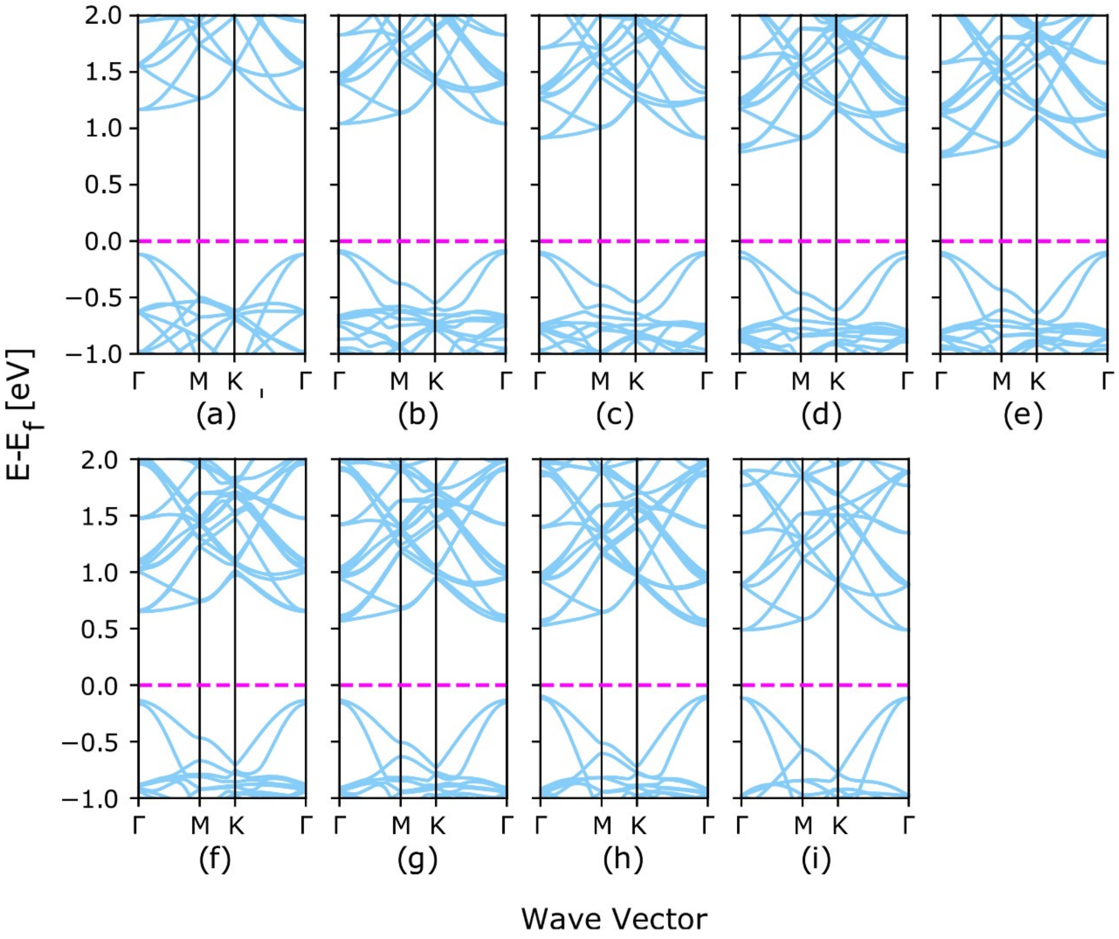

Fig. S1 Folded electronic band-structures of (a) $Hf_{16}S_{32}$ (b) $Hf_{16}S_{28}Se_4$ (c) $Hf_{16}S_{24}Se_8$ (d) $Hf_{16}S_{20}Se_{12}$ (e) $Hf_{16}S_{16}Se_{16}$ (f) $Hf_{16}S_{12}Se_{20}$ (g) $Hf_{16}S_8Se_{24}$ (h) $Hf_{16}S_4Se_{28}$ (i) $Hf_{16}Se_{32}$ in a 4 × 4 × 1 supercell.

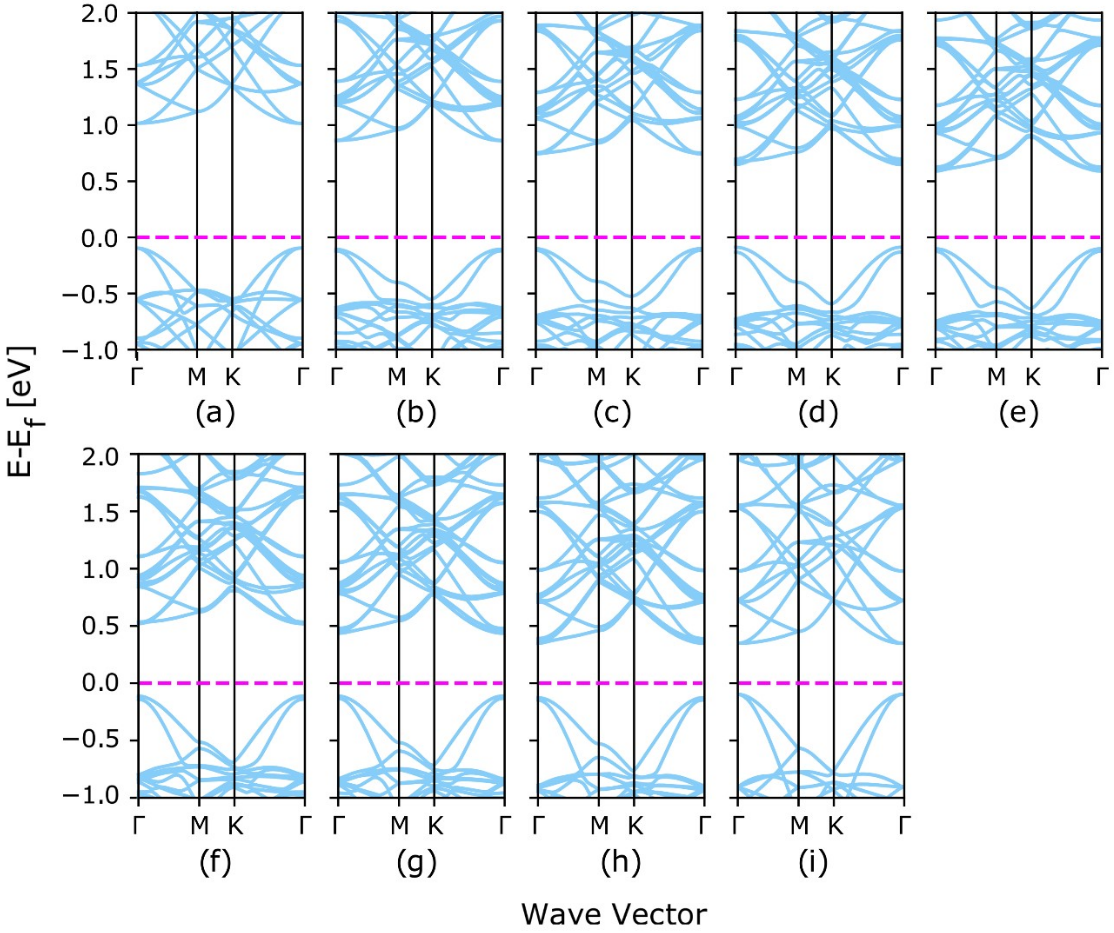

Fig. S2 Folded electronic band-structures of (a) $Zr_{16}S_{32}$ (b) $Zr_{16}S_{28}Se_4$ (c) $Zr_{16}S_{24}Se_8$ (d) $Zr_{16}S_{20}Se_{12}$ (e) $Zr_{16}S_{16}Se_{16}$ (f) $Zr_{16}S_{12}Se_{20}$ (g) $Zr_{16}S_8Se_{24}$ (h) $Zr_{16}S_4Se_{28}$ (i) $Zr_{16}Se_{32}$ in a 4 × 4 × 1 supercell.

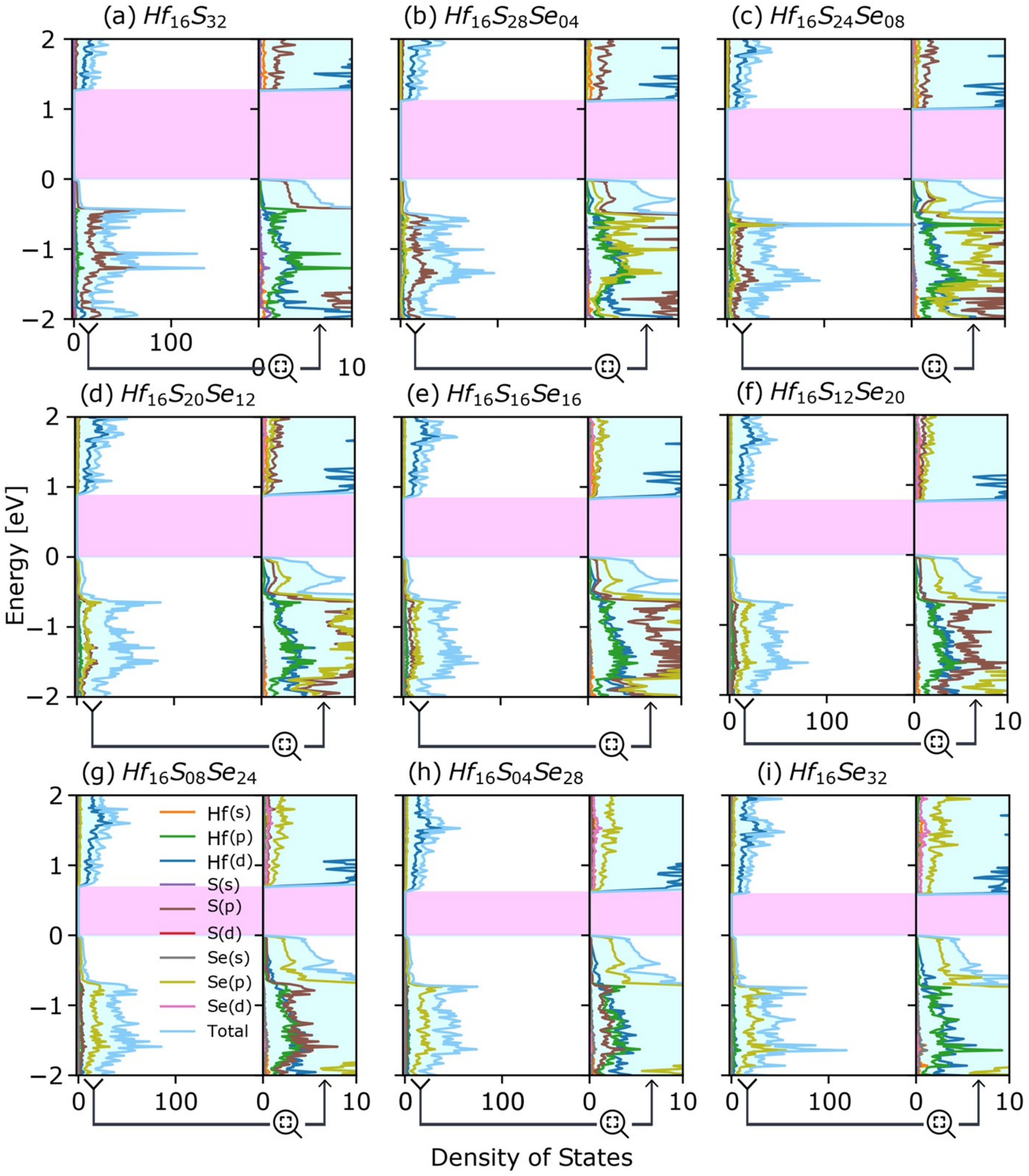

Fig. S3 The total and partial density of states (DOS) of (a) $Hf_{16}S_{32}$ (b) $Hf_{16}S_{28}Se_4$ (c) $Hf_{16}S_{24}Se_8$ (d) $Hf_{16}S_{20}Se_{12}$ (e) $Hf_{16}S_{16}Se_{16}$ (f) $Hf_{16}S_{12}Se_{20}$ (g) $Hf_{16}S_8Se_{24}$ (h) $Hf_{16}S_4Se_{28}$ (i) $Hf_{16}Se_{32}$.

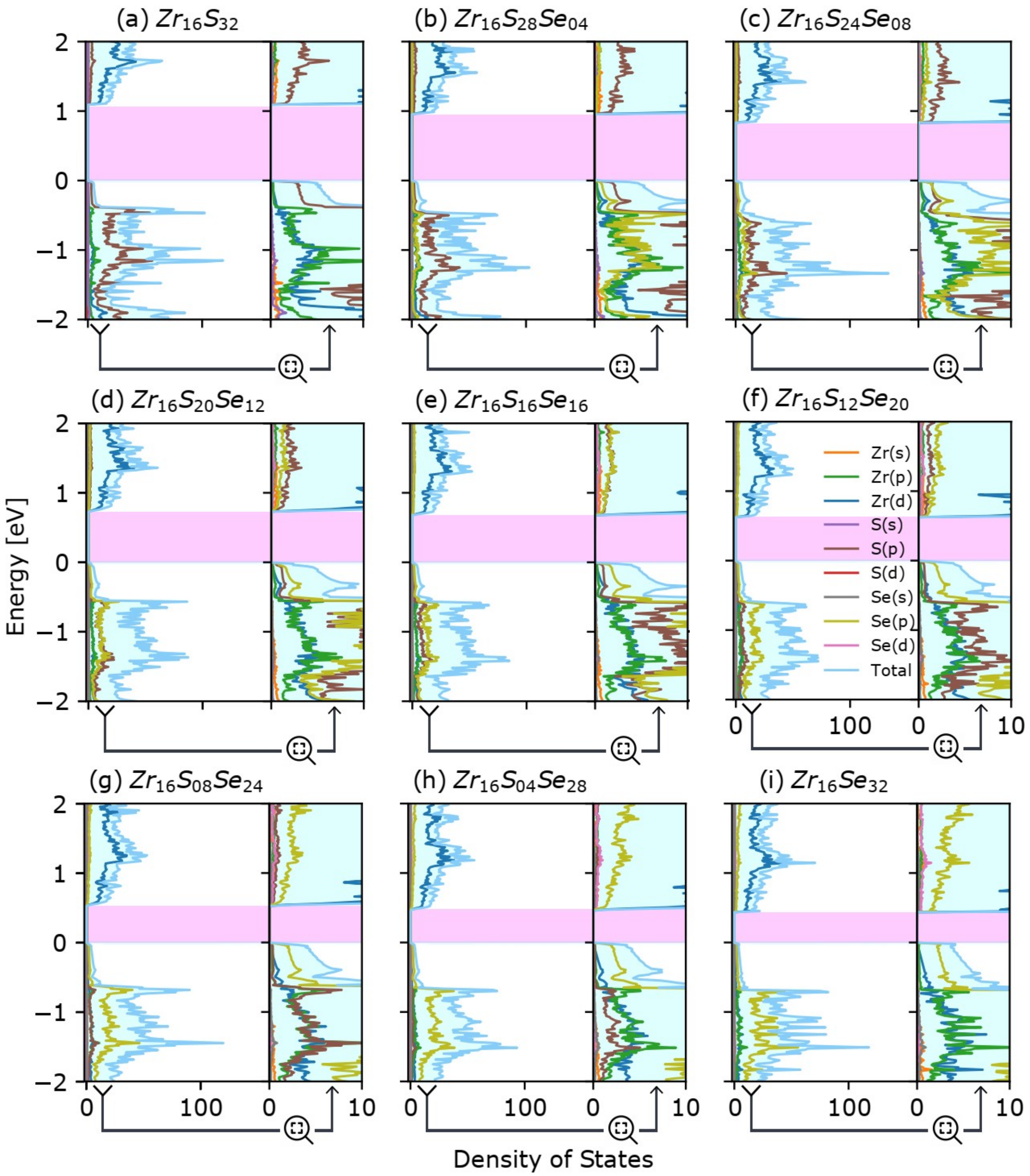

Fig. S4 The total and partial density of states (DOS) of (a) $Zr_{16}S_{32}$ (b) $Zr_{16}S_{28}Se_4$ (c) $Zr_{16}S_{24}Se_8$ (d) $Zr_{16}S_{20}Se_{12}$ (e) $Zr_{16}S_{16}Se_{16}$ (f) $Zr_{16}S_{12}Se_{20}$ (g) $Zr_{16}S_8Se_{24}$ (h) $Zr_{16}S_4Se_{28}$ (i) $Zr_{16}Se_{32}$.
